# Burnout, Presenteeism and Workplace Conditions of Korean Taekwondo Coaches of High-Performance Athletes

**DOI:** 10.3390/ijerph19105912

**Published:** 2022-05-12

**Authors:** Eunchul Seo, Hanbeom Kim, YoungKyun Sim, Min-Seong Ha, Uk Kim, HyunRyun Kim

**Affiliations:** 1 Department of Physical Education, Wonkwang University, 460 Iksan-daro, Iksan 54538, Jeonbuk, Korea; eunchulseo17@wku.ac.kr; 2 Department of Wellness Sports Science, School of Wellness Industry Convergence, Hankyong National University, 327 Jungang-ro, Anseong-si 17579, Gyeonggi-do, Korea; 3 Department of International Sports, Dankook University, 119 Dandae-ro, Dongnam-gu, Cheonan-si 31116, Chungcheongnam-do, Korea; 4 Department of Sports Culture, College of the Arts, Dongguk University, 30 Pildong-ro 1-gil, Jung-gu, Seoul 04620, Korea; haminseong@dgu.ac.kr; 5 Department of Physical Education, Dankook University, 152 Jukjeon-ro, Suji-gu, Yongin-si 16890, Gyeonggi-do, Korea; uk05180@gmail.com; 6 Department of Physical Education, Woosuk University, 443 Samnye-ro, Samnye-eup, Wanju-gun 55338, Jeollabuk-do, Korea; khr0615@naver.com

**Keywords:** taekwondo coach, stress, workplace conditions, burnout syndrome, presenteeism

## Abstract

Coaching is a stressful occupation, with expectations that are physically and psychologically demanding. Coaches are highly susceptible to occupational burnout and presenteeism, which ultimately affects the entire sporting community. In this study, coaching stress was evaluated by surveying taekwondo coaches to analyze the contributions of unique cultural predispositions and workplace conditions (environmental) to coach stress, burnout, and presenteeism. We verified the positive correlation between workplace conditions, burnout, and presenteeism for 210 taekwondo coaches; performed frequency, correlation, and confirmatory analysis using the compiled data; and the discussed the results within the framework of a formulated structural equation model. The research results are as follows. First, the workplace conditions of taekwondo coaches had a negative effect on burnout syndrome. Second, the workplace conditions of taekwondo coaches had a negative effect on presenteeism. Lastly, burnout of taekwondo coaches had a significant effect on presenteeism. Therefore, coaches’ burnout decreases as their workplace conditions improve, and presenteeism decreases as their burnout increases in controlled workplace conditions.

## 1. Introduction

Winning is an inherent objective at all levels of competitive sports. Although it should not be considered the essential core of the games, the weight of a victory can bear significance as it is often used as a direct gauge to measure the success of athletes or coaches at competitive level [1]. At the professional level, where profit generation takes up a large margin of executive considerations, the value of winning can be overbearing, and has a direct economic impact over supporter patronage, media coverage, or corporate sponsorships [2,3,4,5]. Under such profit-oriented leadership, athletes are often exhausted by intense physical and mental demands and are stressed by having to deal with public scrutiny, abrasive group dynamics, and the constant exposure to career-terminating or life-altering injury risks [6]. Furthermore, athletes are burdened by the need to perform at maximum capacity at every given opportunity in the face of inevitable age-dependent physical capabilities, and consequently, relatively short-lived professional careers [7,8,9]. With an increasing number of athletes sharing their struggles with depression during and after their career [10,11,12], mainstream research on sports psychology has focused primarily on the perspective of athletes [13,14]. In contrast, the coaches’ perspective regarding how they are affected by organizational dynamics is less established [14].

Coaching is an inherently stressful occupation. However, a coach’s vulnerability to stress is less ascertained, especially because coaches are expected to be the problem solvers and not the problem bearers [15]. In particular, a distressed coach is often perceived as exhibiting weak leadership and incompetence due to the immediate psychological and demoralizing impact on athletes, which can directly lead to poor performance [16]. Therefore, coaches acknowledge stress as a predesignated condition that comes with the job, and willingly conceal, restrain, and endure stress to fulfill their role in promoting satisfaction and well-being among their athletes [17].

Nonetheless, research has shown that poor stress management and lack of coping strategies for coaches will inevitably lead to instability within the team, which may ruin the delicate coach–athlete relationship, cause a loss of confidence, impair performance, and reduce enthusiasm [18,19]. Various stressors in coaching have been discussed in the literature. For example, Frey conducted a series of interviews with coaches competing in National Collegiate Athletic Association competitions and found a common theme of anxiety with their job, particularly with hardships that involved communication difficulties, lack of control over athletes, talent recruiting, multitasking, lack of personal time, and forced family separation [15]. With high school athletic coaches, Sage noted a prevalence of job burnout over perceived complexity and work overload, and observed individuals struggling between coaching and teaching priorities [20]. Yet, even Olympic coaches, who work with athletes of the highest caliber were found to suffer from anxiety, stress, and exhaustion with regard to self- or peer-evaluated criticisms of their coaching efficacy and the coach–athlete relationship (e.g., gaining trust, handling crises, staying composed under pressure, making critical decisions) [21].

Irrespective of the circumstances, coaches’ obligations are easily overextended to support athlete-centered causes, which require coaches to fill the position of an instructor, mentor, friend, organizer, educator, or counselor [22]. Despite the intricate interpersonal nature of the job, coach-training programs are fundamentally designed to promote athletic achievements [23]. At the end of the day, a coach’s productivity is assessed on the basis of the success of athletes or results of the competitions [24]. Hence, coaches who experience high levels of stress are unsurprisingly common considering the mismatch between the misguided objectives and the expectations imposed on their roles, and the shortfall of organizational support relative to their bestowed responsibilities and liabilities. Although there is a growing awareness regarding the topic of coaching stress, often empathy is aligned with the consequences of athletic performance and team dynamics rather than the well-being of the affected individuals [18,25]. In this regard, Olugosa urged the need for a comprehensive study of coaching stress from the perspective of the coaches, which would be contingent on reviewing the origins of each stressor, and a thorough examination of the short- and long-term effects and the positive and negative impacts of stress on each individual beyond the scope of the overly simplified stress and burnout models [24].

Occupational burnout syndrome is frequently paired with employees who feel overwhelmed and impaired with stress in the workplace. Professions that function in a provider–recipient dynamic, such as teachers, doctors, nurses, people in the police force, and hair designers, have been shown to be prone to stress and burnout and frequently sought out for job-burnout-related studies [26]. First coined by Hubert Freudenberger in 1974 [27], the term “job burnout” has been further embraced by Maslach, Schaufeli, and Leiter to describe a state of emotional exhaustion that typically leads to cynicism and interpersonal disengagement, and eventually to an extensive detachment from the various aspects of the job [28]. In particular, Maslach et al. [28] developed Freudenberger’s burnout definition more specifically to “a status of emotional burnout, depersonalization, and reduced personal accomplishment”.

Burnout has been cited as the root of pessimism toward coworkers, the company, the assignments; and analogously in a sports team, the cause of discord among teammates and between athletes and coaches. For coaches at the high-performance level, Lundkvist was able to identify soccer coaches fitting the description of being burned out due to work overload, pressure from sports’ performance culture, and a lack of tools for stress management and recovery [29]. Coaches commonly claim that irregular work hours, overwhelming workloads, and unmatched benefits also impede motivation and productivity and have a negative impact on work–life balance [30,31]. According to an article from the Harvard Business Review [32], an estimated USD125–USD190 billion is spent every year on healthcare expenses for the treatment of work-related stress and burnout. However, whether “burnout syndrome” is a diagnosable medical condition has been heavily debated [33]. Recently, with the release of the 11th edition of the International Classification of Diseases from the World Health Organization, burnout has gained recognition as an occupational phenomenon; however, it is yet to be defined as a mental disorder [34]. There have been other efforts, such as company policies to create guidelines for stress management and infrastructure to support recovery from burnout. However, for coaches, cultural displacement within the sports industry makes a case of burnout an illegitimate argument for any absence. Regardless, the intricate coach–athlete relationship makes it nearly impossible for coaches to remove themselves from their posts; for such reasons, a high prevalence of coaches working through exhaustion or poor physical/mental conditions is expected, otherwise known as presenteeism [35,36].

Presenteeism is defined as a phenomenon in which job performance is negatively affected or attention is lost when performing a job in poor condition [37]. Stewart et al. [38] argued that it is a physical and psychological degradation caused by negative work environment. The stress and presenteeism experienced by taekwondo coaches of high-performance athletes can result in burnout. In recent years, burnout has easily been found in various occupational groups [39]. It was reported that Korean office workers also suffer from burnout due to mental stress and chronic fatigue [40]. Lee et al. [41] reported that burnout occurred due to excessive job stress. Moreover, many studies have consistently reported claims that burnout affects presenteeism [38].

Most of the past research has not defined stress and burnout, and although the aftereffects of burnout have been superficially discussed, a thorough examination of the specific issues, such as presenteeism, that directly impact the quality of the experience of the athletes and coaches is lacking. Therefore, this study aims to analyze the relationship between the source of stressors, with coach burnout and presenteeism as the main variables, and identify their correlations.

In this study, we selected Korean taekwondo coaches of high-performance athletes as participants to construct our coaching stress model, in consideration of the overarching prestige of the sport due to Korea being the country in which taekwondo originated. We were especially interested to observe how athletes and coaches are affected by the pressure to exert and maintain global dominance. We hypothesized that cultural influences would contribute significantly to shaping the organizational infrastructure, which would consequently outline the standards for coaching. We defined workplace conditions to be factored by organizational guidance, financial stability, facility quality, and athlete quality, and hypothesized that workplace conditions would be a stressor for coaches, which would correlate with occupational burnout and presenteeism.

**Figure 1:**
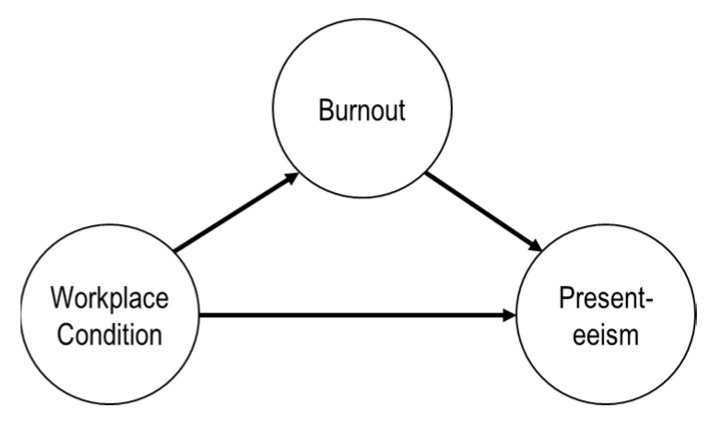
The research model.

Therefore, the purpose of this study was to investigate the relationship between the burnout, presenteeism and workplace conditions in Korean taekwondo coaches of high-performance athletes. Through this, the researchers intended to provide basic data for improving the status of Korean taekwondo coaches of high-performance athletes, and improving their level of welfare. The research hypotheses and model (Figure 1) were as follows.

**Hypothesis** **1.**  *The workplace conditions for Korean taekwondo coaches of high-performance* *athletes will* *have* *a* *negative* *correlation* *with ut syndrome.*

**Hypothesis** **2.**  *The workplace conditions for taekwondo coaches will have a negative relationship with presenteeism.*

**Hypothesis** **3.**  *The burnout of taekwondo coaches will have a positive relationship with presenteeism.*

## 2. Materials and Methods

### 2.1. Participants and Procedure

This study complied with STROBE (Strengthening the Reporting of Observational Studies in Epidemiology) checklist. The participants of this study were selected from taekwondo coaches of high-performance athletes who took part in the Korean Annual National Athletics Competition and/or the National Minister of Defense Taekwondo Competition (from 8 October 2021 to 14 October) in South Korea. With the cooperation of the organizers, we asked all the coaches who participated in the competition to take the survey. Among them, coaches who agree to participate were selected as research participants.

All coaches worked with taekwondo athletes performing competitively at a high performance level in South Korea, and had been coaching for over 5 years. All taekwondo coaches had their own training center, and trained athletes at the training center. Coaches who participated in this study were informed about aim of this study and confidentiality. They answered a set of questionnaires voluntarily, which took approximately 15 min to complete. From among data from a total 218 coaches, 8 were omitted due to lack of consistency or missing data; the remaining 210 surveys were used for data processing. The demographics of the participants are presented in Table 1.

The survey was conducted on-site at the 99th Annual National Athletics Competition and 27th National Minister of Defense Taekwondo Competition, by the investigator, accompanied by two assistants. The survey was conducted with permission from the event committee. A survey was conducted on all coaches who participated in the competition, except for coaches who refused to participate in the study. During the competition, investigators individually met with coaches and explained the purpose of the research and survey, and coaches filled out a questionnaire in a nearby office or in a space with a table. A total of 218 taekwondo coaches of high-performance athletes participated in the self-assessment survey, and the completed forms were retrieved by the investigators.

### 2.2. Measures

This study adopted a survey questionnaire that was used in a previous study [37,41,42]. The survey consisted of questions relating to the participants’ personal data, which were broken down into four different categories: demographics, workplace conditions, burnout syndrome, and presenteeism.

#### 2.2.1. Workplace Conditions

The 12 questions relating to workplace conditions were modeled after the validated survey from Jeon et al. [42] which included work relationships (3 questions), pay grade (3 questions), work environment (3 questions), and welfare benefits (3 questions). Validity tests on the Likert scale revealed that 3 out of the 12 questions (question numbers 4, 8, and 12) did not meet unidimensionality and were removed. The goodness-of-fit was measured by the Pearson’s chi-square test, returning the following results: chi-square (χ^2^) = 50.652 (*p* < 0.001), degree of freedom (df) = 21, confirmatory fit index (CFI) = 0.953, Tucker Lewis Index (TLI) = 0.920, and root mean square error of approximation (RMSEA) = 0.080. Our tests confirmed that the measured goodness was acceptable for modeling. The standardized regression coefficient was >0.562, and Cronbach’s coefficient α was >0.70, verifying the reliability.

#### 2.2.2. Burnout Syndrome

The burnout syndrome category was adopted from a prior Coach Burnout Affect Survey [41]. It consisted of 20 inquiries, including physical deprivation (4 questions), poor workplace conditions (4 questions), unfair treatment (4 questions), and casual discord (4 questions). Validity tests on the Likert scale in the burnout syndrome responses revealed that 6 out of the 16 questions (question numbers 4, 8, 11, 12, 14, and 20) did not meet unidimensionality and were removed. The revised version showed a goodness-of-fit of χ^2^ = 151.029 (*p* < 0.001), df = 67, CFI = 0.933, TLI = 0.908, and RMSEA = 0.077, confirming its validity. The standardized regression coefficient and Cronbach’s coefficient α were >0.624 and >0.70, respectively, verifying the reliability of the finalized list of questions.

#### 2.2.3. Presenteeism

Questions relating to presenteeism used the Stanford Presenteeism Scale, which was originally developed by Turpin et al. [37], It consisted of 10 inquiries, completing work (5 questions), Avoiding Distraction (5 questions). Validity tests on the Likert scale revealed that 2 out of the 10 questions (question numbers 5, and 9) did not meet unidimensionality and were removed. The revised version showed a goodness-of-fit of χ^2^ = 34.54 (*p* < 0.001), df = 19, CFI = 0.987, TLI = 0.980, and RMSEA = 0.063, confirming its validity. The standardized regression coefficient and Cronbach’s coefficient α were >0.643 and >0.80, respectively, verifying the reliability of the questions.

### 2.3. Data Analysis

The collected data were processed using IBM SPSS 23.0 and AMOS 23.0. The null hypothesis was verified and the significance level (α) was set to 0.05. The details of the analytical methods are as follows.

To resolve the demographics of the sample pool, frequency and correlation analyses were performed. Normality was measured by skewness and kurtosis, which referenced West et al. for the critical values [43]. In accordance with West and colleagues, the values for skewness and kurtosis were standardized at ±2 and ±4, respectively, from the critical value, and any test results in excess were considered unreliable. Additionally, confirmatory factor analysis was conducted with the incorporation of the ML method, and Cronbach’s coefficient α reliability testing was performed. For the goodness-of-fit, a chi-square test was performed to retrieve the values for χ^2^, df, CFI, TLI, and RMSEA. To test the original hypothesis, a structural equation model (SEM) was implemented, which verified the accuracy of the modeling through a 2-step method that required the validation of a measurement model before formulating the SEM [44]. Finally, bootstrapping simulation with a sample size of 2000 and a bias-corrected confidence interval of 95% was performed to verify the significance of the indirect influence of burnout syndrome on workspace conditions and presenteeism.

The questionnaire was administered to a group of professionals (two taekwondo professors, one taekwondo PhD, and two taekwondo coaches of high-performance athletes) to verify the validity and goodness. The validity of the analytical methods was tested by confirmatory factor analysis based on the maximum likelihood (ML) method, and Cronbach’s coefficient α was calculated for reliability. The details of the confirmatory factor analysis and reliability results are shown in Table 2.

## 3. Results

### 3.1. Correlation Analysis of Variables

This study aimed to investigate the correlations between the three major variables: workplace conditions, burnout syndrome, and presenteeism. The detailed results are presented in Table 3. In brief, the subvariables under each category demonstrated partial correlativity, while the probability for multicollinearity was negligible, with all estimated coefficients recording less than the multicollinearity threshold value of 0.80 [45].

### 3.2. Normality Test

Considering that the measurement model and SEM were implemented using the ML method, a multivariate normality test was conducted to validate the null hypothesis. Generally, when multivariate normality is valid, it is likely that the normality of the models would also be significant. Multivariate normality was tested by evaluating the skewness and kurtosis of the fitted model, and a range of 0.858 to 1.074 was recorded for skewness, while a range of −0.673 to 1.986 was observed for kurtosis. The results were within the range of ±2 and ±4, respectively, for the two values. As defined by West et al., the results were determined to satisfy the normality test. The details of the normality test are presented in Table 4.

### 3.3. Measurement Model Test

As recommended by Anderson and Gerbing [44], the measurement model was tested prior to the SEM evaluation. The chi-square test results for the measurement model revealed excellent goodness-of-fit with the specific values as follows: χ^2^ = 125.152, df = 0.51, TLI = 0.900, CFI = 0.922, and RMSEA = 0.079 (Table 5). In addition, all standardized regression coefficients exceeded 0.482, which suggested that the measured variables were within a reasonable fit. With confirmation of the goodness of the measurement model [46], the validity of the SEM was subsequently tested according to the guidelines by Anderson and Gerbing [44].

### 3.4. Structural Equation Model Test

To determine the statistical significance of the research hypothesis, a statistical model was established, which was tested by the SEM. An elite taekwondo instructor’s workplace condition was set as the exogenous variable, the correlative presenteeism was designated as the endogenous mediator variable, and the burnout syndrome as the endogenous dependent variable. Based on the chi-square test results with values of χ^2^ = 125.152, df = 0.51, TLI = 0.900, CFI = 0.922, and RMSEA = 0.079, the hypothesized SEM was proven to be valid. With a validated SEM, the hypothesized correlations between the main variables were analyzed along with the corresponding path coefficients, as shown in Table 6.

The taekwondo coaches’ workplace condition was found to be positively correlated with the likelihood of experiencing burnout syndrome (β = −4.622, *p* < 0.001) and significantly positively correlated with presenteeism (β = −2.631, *p* < 0.01). Lastly, the impact of burnout syndrome was negatively correlated with the prevalence of presenteeism (β = 2.172, *p* < 0.05) (Table 6). To further examine the mediating effects of burnout syndrome, statistical significance was examined via a bootstrapping simulation with a sample size of 2000 and a bias-corrected confidence interval of 95%. The resulting lower and upper bounds were 0.011 and 0.216, respectively, and the *p*-value was found to be statistically significant (*p* < 0.05). In summary, the results revealed that with greater challenges or increased confinement within the workplace, there is an increase in the likelihood of experiencing burnout and, correspondingly, a reduction in presenteeism. It can be interpreted that as burnout increases, presenteeism decreases.

## 4. Discussion

This study investigated the impact of workplace conditions on burnout rate and presenteeism among taekwondo coaches of high-performance athletes. The purpose of this investigation was to provide a data-driven referendum to support and advocate for the need for an appropriate adjustment to the status quo of taekwondo coaches of high-performance athletes. The key findings of the correlative analyses of the main variables are discussed below.

First, it was found that the workplace condition of taekwondo coaches of high-performance athletes was in negative relationship with levels of burnout. Namely, self-assessed quality evaluations of workplace conditions were inversely proportional to work-related burnout. This corroborated previous investigations linking employee burnout to workplace conditions and agreed with a research report that demonstrated a correlative decrease in burnout occurrences with workplaces scoring high in workplace conditions [46,47,48]. Various factors within the workplace can dictate the exposure level to burnout risk, especially for taekwondo coaches of high-performance athletes working on a part-time or contractual basis, who need to rely on establishing and maintaining compliance within the school, with the athletes, and with the parents, for job security. Such circumstances demand that coaches prioritize the organization’s needs before their own personal needs; hence, it may make them especially vulnerable to burnout. To appropriately address the mismatch, a practical solution needs to be developed that will optimize workplace conditions, lessen the burden for sports coaches of high-performance athletes, and enable optimal output, which will eventually translate into improved productivity and performance.

Second, the workplace conditions of taekwondo coaches of high-performance athletes were in negative relationship with presenteeism. Assessments of workplace conditions were inversely linked to presenteeism. This is in line with several other studies that identify workplace conditions as a key factor for predicting presenteeism, and verifies the claim that presenteeism will reduce with improvements in the workplace and increase when the workplace is resentful [49,50]. As such, an enabling atmosphere is expected to contribute to higher self-esteem, which will likely lead to an overall positive output when interacting and communicating with athletes. Our results suggested that adequate on-site support and worksite improvements are needed to prevent unwarranted inconveniences that can hinder job performance and cause mental or physical issues; these may lead to presenteeism.

Lastly, burnout was in positive relationship with presenteeism. The occurrences of burnout syndrome and presenteeism in taekwondo coaches of high-performance athletes were found to be positively correlated. This study supported previous findings that investigated the effect of burnout in employees as it related to the prevalence of presenteeism [51,52]. Choi and Jeong identified burnout syndrome as an influential factor on presenteeism [35], and Hwang claimed that disposition to burnout triggers presenteeism and causes a decline in worksite performance [53]. Additionally, depression, anxiety, and lethargy are commonly associated with burnout, which can lead to health issues, especially in individuals such as taekwondo coaches, who hold occupations that require constant high-intensity engagement. Therefore, prevention of burnout is recommended, which can be achieved through simple morale boosts, such as financial incentives or improved retirement benefit policies.

The limitation of this study was that it conducted from a limited pool of taekwondo coaches who were present at the Korean Annual National Athletics Competition and the National Minister of Defense Taekwondo Competition in Korea. Since this study mainly targeted taekwondo coaches of high-performance athletes, it is difficult to apply the research results to taekwondo coaches who coach other levels (e.g., youth taekwondo education, low-level taekwondo training programs, etc.).

## 5. Conclusions

An analysis of 210 surveys gathered from taekwondo coaches of high-performance athletes who participated in the Korean Annual National Athletics Competition and/or the National Minister of Defense Taekwondo Competition in Korea, was conducted to examine the effect of the workplace environment on the prevalence of burnout syndrome and presenteeism among the participants.

It was found that taekwondo coaches of high-performance athletes experienced less burnout and presenteeism in a positive workplace condition. This suggests that there is a need to improve the workplace condition of coaches, to improve their work performance. In other words, it can be effective to improve their workplace condition to achieve better performance. Unfortunately, in reality, efforts to improve workplace conditions seem to be largely insufficient. Additionally, the more burnout appears, the more they experience presenteeism. This suggests that new policy is needed to prevent coaches from experiencing burnout.

Workplace conditions have a large influence on the performance of a taekwondo coach. Therefore, in order for coaches to perform effective coaching, it seems necessary to improve workplace conditions. Through this study, it was possible to derive the conclusion that it is necessary to create a positive environment for taekwondo coaches of high-performance athletes, so that coaches can effectively perform their role. The authors consider it is time for the creation of policy to prevent them from experiencing burnout and presenteeism, through an improvement of their workplace conditions.

## Figures and Tables

**Table 1:** Demographics of Participants.

Demographics	Category	Frequency	(%)
Gender	Male	151	71.9
	Female	59	28.1
Age	20s	28	13.3
	30s	145	69.0
	40+	37	17.6
Affiliation	Middle School	69	32.9
	High School	70	33.3
	University	71	33.8
Duration	6~7 years	70	33.3
	7~9 years	83	39.6
	Over 10 years	57	27.1
Total		210	100

**Table 2:** Confirmatory factor analysis of each potential variable and reliability results.

Latent Variable		*m* Variable	B	β	S.E	T	a	Goodness
Workplace Conditions	Work relationship	a1	1.000	0.718			0.794	χ^2^ = 50.652 df = 21 TLI = 0.920 CFI = 0.953 RMSEA = 0.080
		a2	1.358	0.822	0.137	9.625 ***		
		a3	1.331	0.740	0.124	9.205 ***		
	Pay grade	a5	1.000	0.866			0.769	
		a6	0.776	0.723	0.102	8.587 ***		
	Work environment	a7	1.000	0.562			0.739	
		a9	1.781	0.891	0.280	6.357 ***		
	Welfare Benefits	a10	1.000	0.795			0.779	
		a11	0.925	0.739	0.107	8.637 ***		
Burnout Syndrome	Physical Deprivation	b1	1.000	0.724			0.822	χ^2^ = 151.029 df = 67 TLI = 0.908 CFI = 0.933 RMSEA = 0.077
		b2	1.469	0.840	0.135	10.884 ***		
		b3	1.222	0.782	0.118	10.330 ***		
	Mental Deprivation	b5	1.000	0.696			0.815	
		b6	1.347	0.824	0.136	9.905 ***		
		b7	1.171	0.788	0.121	9.678 ***		
	Poor workplace conditions	b9	1.000	0.790			0.726	
		b10	.923	0.721	0.105	8.806		
	Unfair treatment	b13	1.000	0.640			0.720	
		b15	1.321	0.695	0.168	7.873 ***		
		b16	1.276	0.708	0.160	7.972 ***		
	Casual Discord	b17	1.000	0.624			0.754	
		b18	1.674	0.836	0.231	7.235 ***		
		b19	1.494	0.740	0.215	6.953 ***		
Presenteeism	Completing work	c1	1.000	0.913			0.884	χ^2^ = 34.54, df = 19 TLI = 0.980 CFI = 0.987 RMSEA = 0.063
		c2	1.345	0.917	0.125	10.766 ***		
		c3	1.317	0.783	0.122	10.785 ***		
		c4	1.215	0.643	0.126	9.657 ***		
	Avoiding distraction	c6	1.000	0.718			0.904	
		c7	1.233	0.931	0.094	13.145 ***		
		c8	1.341	0.924	0.103	13.075 ***		
		c10	1.095	0.800	0.096	11.349 ***		

*** *p* < 0.001.

**Table 3:** Analysis of correlation between variables.

	1	2	3	4	5	6	7	8	9	10	11
1	1										
2	0.231 **	1									
3	0.262 **	0.166 *	1								
4	0.406 **	0.353 **	0.381 **	1							
5	−0.208 **	−0.236 **	−0.024	−0.137 *	1						
6	−0.152 *	−0.140 *	−0.032	−0.166 *	0.561 **	1					
7	−0.233 **	−0.410 **	−0.072	−0.307 **	0.519 **	0.423 **	1				
8	−c0.229 **	−0.169 *	−0.098	−0.177 *	0.519 **	0.403 **	0.505 **	1			
9	−0.300 **	−0.211 **	−0.204 **	−0.227 **	0.433 **	0.363 **	0.474 **	0.596 **	1		
10	−0.305 **	0.019	−0.006	−0.332 **	0.111	0.215 **	0.206 **	0.227 **	0.166 *	1	
11	−0.396 **	−0.194 **	−0.102	−0.400 **	0.367 **	0.387 **	0.517 **	0.547 **	0.653 **	0.374 **	1

Note. 1 = work relationship, 2 = pay grade, 3 = work environment, 4 = welfare benefits, 5 = physical deprivation, 6 = mental deprivation, 7 = poor workplace conditions, 8 = unfair treatment, 9 = casual discord, 10 = completing work, 11 = avoiding distraction, * *p* < 0.05, ** *p* < 0.01.

**Table 4:** Confirmatory factor analysis of each potential variable and reliability results.

Item	Skewness		Kurtosis	
	S	SEM	S	SEM
Work environment	−1.031	0.168	1.986	0.334
Work relationship	0.579		0.221	
Pay grade	−0.083		0.231	
Welfare benefits	−0.086		−0.025	
Phys/mental deprivation	−0.327		0.257	
Casual discord	−0.530		0.423	
Poor workplace conditions	−0.204		−0.673	
Unfair treatment	−0.151		−0.436	
Presenteeism	−0.249		−0.598	
Completing Work	0.791		0.078	
Avoiding Distraction	0.138		−0.418	

**Table 5:** Measurement model path.

Latent variable	*m* Variable	B	β	t
Workplace Conditions	Welfare benefits	1.000	0.872	
	Pay grade	0.541	0.482	6.133 ***
	Work environment	0.496	0.558	6.975 ***
	Welfare benefits	1.428	0.872	6.140 ***
	Welfare benefits	0.544	0.574	7.143
Burnout Syndrome	Physical deprivation	1.000	0.577	
	Mental deprivation	1.116	0.577	7.196 ***
	Poor workplace conditions	1.394	0.754	8.937 ***
	Unfair treatment	1.351	0.695	8.392 ***
	Casual discord	1.328	0.756	8.959 ***
Presenteeism	Completing work	1.000	0.710	-
	Avoiding distraction	1.018	0.914	19.701 ***
χ^2^ = 125.152, df = 51, TLI = 0.900, CFI = 0.922, RMSEA = 0.079				

*** *p* < 0.001.

**Table 6:** Structural equation model (SEM) analysis result.

Variable		Variable	B	β	t	SMC	Result
Workplace environment	→	Burnout	−0.257	−0.490	−4.622 ***	0.241	Accept
Workplace environment	→	Presenteeism	−0.176	−0.250	−2.631 **	0.162	Accept
Burnout	→	Presenteeism	0.291	0.217	2.172 *		Accept
χ^2^ = 125.152, df = 51, TLI = 0.900, CFI = 0.922, RMSEA = 0.079							

* *p* < 0.05, ** *p* < 0.01, *** *p* < 0.001.
